# Risk-Reducing Bilateral Salpingo-Oophorectomy for BRCA Mutation Carriers and Hormonal Replacement Therapy: If It Should Rain, Better a Drizzle than a Storm

**DOI:** 10.3390/medicina55080415

**Published:** 2019-07-29

**Authors:** Maria Luisa Gasparri, Katayoun Taghavi, Enrico Fiacco, Veronica Zuber, Rosa Di Micco, Guglielmo Gazzetta, Alice Valentini, Michael D. Mueller, Andrea Papadia, Oreste D. Gentilini

**Affiliations:** 1 Breast Surgery Unit, San Raffaele University Hospital, via Olgettina 60, 20132 Milan, Italy; 2 Institute of Social and Preventive Medicine, University of Bern, Mittelstrasse 43, 3012 Bern, Switzerland; 3 Department of Clinical medicine and Surgery, University of Naples Federico II, Corso Umberto I 40, 80138 Naples, Italy; 4 Department of Obstetrics and Gynecology, University Hospital of Bern, Friedbühlstrasse 19, 3010 Bern, Switzerland; 5 Department of Obstetrics and Gynecology, University of the Italian Switzerland (USI) and Ente Ospedaliere Cantonale (EOC), Via Tesserete 46, 6900 Lugano, Switzerland

**Keywords:** bilateral salpingo-oophorectomy, BRCA mutation carriers, breast cancer, hormonal replacement therapy, menopause, ovarian cancer, risk reducing surgery

## Abstract

Women carrying a BRCA mutation have an increased risk of developing breast and ovarian cancer. The most effective strategy to reduce this risk is the bilateral salpingo-oophorectomy, with or without additional risk-reducing mastectomy. Risk-reducing bilateral salpingo-oophorectomy (RRBSO) is recommended between age 35 and 40 and between age 40 and 45 years for women carriers of BRCA1 and BRCA2 mutations, respectively. Consequently, most BRCA mutation carriers undergo this procedure prior to a natural menopause and develop an anticipated lack of hormones. This condition has a detrimental impact on various systems, affecting both the quality of life and longevity; in particular, women carrying BRCA1 mutation, who are likely to have surgery earlier as compared to BRCA2. Hormonal replacement therapy (HRT) is the only effective strategy able to significantly compensate the hormonal deprivation and counteract menopausal symptoms, both in spontaneous and surgical menopause. Although recent evidence suggests that HRT does not diminish the protective effect of RRBSO in BRCA mutation carriers, concerns regarding the safety of estrogen and progesterone intake reduce the use in this setting. Furthermore, there is strong data demonstrating that the use of estrogen alone after RRBSO does not increase the risk of breast cancer among women with a BRCA1 mutation. The additional progesterone intake, mandatory for the protection of the endometrium during HRT, warrants further studies. However, when hysterectomy is performed at the time of RRBSO, the indication of progesterone addition decays and consequently its potential effect on breast cancer risk. Similarly, in patients conserving the uterus but undergoing risk-reducing mastectomy, the addition of progesterone should not raise significant concerns for breast cancer risk anymore. Therefore, BRCA mutation carriers require careful counselling about the scenarios following their RRBSO, menopausal symptoms or the fear associated with HRT use.

Historically, menopause was considered to be a form of neurosis resulting from the loss of femininity and until 1980, menopause was included among the criteria defining psychosis in the Diagnostic and Statistical Manual of Mental Disorders (DSM). The psychoanalytic theory which underpins this diagnosis relates to that of female sexual development and according to Freud’s theory, women may face depression at this time which can be explained by the female castration complex.

Nowadays, according to the National Comprehensive Cancer Network (NCCN) guidelines, risk-reducing bilateral salpingo-oophorectomy (RRBSO) is recommended after completion of childbearing between age 35 and 40 years for women carriers of BRCA1 mutations and between age 40 and 45 years for women carriers of BRCA2 mutations in order to reduce their risk of developing breast and ovarian cancer. It is estimated that 65% of women carrying BRCA1 mutation will have risk-reducing salpingo-oophorectomy prior to their natural menopause [1], experiencing an earlier onset of surgically induced menopause.

The beneficial effects of an RRBSO include a reduction in ovarian cancer incidence of up to 96% and in breast cancer incidence up to 50% [2,3]. This translates to a reduction in ovarian cancer-specific mortality of 95% and in breast cancer-specific mortality of 42% [4]. Interestingly, the risk reduction in breast cancer-specific mortality is more pronounced for BRCA1 mutated women (HR 0.45, *p* < 0.0001) as compared to BRCA2 mutated women for whom the reduction in breast cancer-specific mortality loses significance (HR 0.88, *p* = 0.75) [4].

The drawback of such a strikingly effective strategy in reducing the risk to develop a potentially deadly cancer, such as ovarian cancer is an earlier onset of menopause with subsequent associated long-term health consequences [5].

In the general population, menopause occurs at a median age of 52 and the onset of menopause at an age younger or equal to 40 years, in gynecology, is defined as premature ovarian failure.

The lack of hormones that characterizes this status involves various systems and may affect both quality of life and longevity. Consequently, in women incurring premature ovarian failure, according to the European Society of Human Reproduction and Embryology (ESHRE) guidelines, hormonal replacement therapy should be started at the time of diagnosis. This should be continued until the average age at which the menopause occurs in the general population as a prevention measure against menopausal symptoms, cardiovascular diseases, osteoporosis, and to reduce the long-term morbidity associated with the hormone depletion [6].

The most characteristic menopausal symptoms, which result from estrogen deprivation and affecting more than 80% of women, are the hot flushes, which can significantly impair the quality of life [7].

Following premature ovarian failure, 43–50% of the women report low sexual desire, over 50% of the women report vaginal dryness, 17–42% report sexual discomfort and/or pain, and 60% report difficulties in reaching an orgasm [8]. To relieve these genitourinary symptoms, lubricants and moisturizers are often prescribed to no avail, whereas the most efficient strategy is a topical treatment with estrogens (17-beta-estradiol, conjugated equine estrogens, or estriol) [9,10].

In addition to these symptoms, neurological dysfunction, cognitive impairment, and dementia are reported in several cases [11,12]. Despite the lack of strong data supporting the benefits of hormonal replacement therapy on memory and communication, ESHRE guidelines recommend the adoption of hormonal replacement therapy in women with premature ovarian failure to reduce the risk of cognitive impairment [6]. With regards to mood disorders including depressive symptoms, there is evidence suggesting the beneficial effects of a hormonal replacement therapy [13].

Given the crucial role played by the estrogens in the metabolism of the bones, it is clear that the early onset of menopause has detrimental effects on the bone density and bone structure leading to an increased risk of bone fracture [5,14]. The negative effect of early-onset menopause on bone mineral density reduction has been recorded in the lumbar spine and femur [15]. It has been demonstrated that hormonal replacement therapy can protect against osteopenia and osteoporosis and can restore bone density in women with premature ovarian failure [16]. The protective effect appears to be higher in women who have undergone hormonal replacement therapy for at least five years and is optimized when a combination of transdermal estradiol and vaginal progesterone are used [17].

Finally, the lack of estrogens in women with premature ovarian failure leads to a modification of the lipid profile that leads to endothelial dysfunction with an increased risk of early cardiovascular-related mortality [16,18]. When a hormonal replacement therapy is initiated, the endothelial function can be restored within six months [19]. As such, ESHRE recommends hormonal replacement therapy in women with premature ovarian failure to reduce the risk of cardiovascular disease [6]. Hormonal treatment should always be accompanied with advice on optimizing lifestyle factors, including regular physical activity, a balanced diet, and a cessation of nicotine [6].

Although hormone replacement in women with premature ovarian failure may occur via combined oral contraceptives or via conventional hormonal replacement therapy, the latter method more closely mimics the physiological concentrations of both estrogen and progesterone [18].

Estrogens can be administered orally, transdermally, or topically. The preferred route of administration is the transdermal as it guarantees a systematic distribution of the hormone and is not associated with an increased risk of cardiovascular morbidity linked to the oral administration of the estrogens. The risk of endometrial cancer is 4.5 times greater among women exposed to estrogen therapy without combined progesterone therapy [20]. The systemic administration of estrogens has to be balanced by a course of progestins for at least 10 days per month to prevent endometrial hyperplasia and cancer [21].

Progestins can be administered orally, vaginally, or via a medicated intrauterine device. At present, it is unclear whether the administration of progestins via subcutaneous implants and intramuscular injections imparts sufficient endometrial protection during hormonal replacement therapy. Among the various progestins, it seems that micronized progesterone has a greater cardiovascular protective effect and may reduce the risk of breast cancer risk as compared with other progestins [6]. Although several studies have demonstrated that micronized progesterone confers adequate endometrial protection, some authors still question its efficacy [13,22]. With regards to side effects, micronized progesterone and the most commonly used progestin, medroxyprogesterone acetate, are associated with an increased risk of thrombosis when adopted in hormonal replacement therapy [23].

Despite ESHRE recommendations and the apparent benefits of hormone replacement therapy, approximately 40% of women with premature ovarian failure discontinue treatment within one year [17,24].

Two of the largest studies of HRT raised concerns regarding the safety of HRT in general population, in terms of risk of breast cancer and heart disease [25,26]. The results of these studies received wide publicity, generating panic and restrictions from several regulatory authorities. However, these studies were biased since women included in the studies were mostly in their mid-sixties, often overweight, and thus unrepresentative of women for whom HRT should be considered suitable. Unfortunately, this about turn and retraction of some of the previous findings has received smaller emphasis from the media, therefore, facilitating the persistence of misconceptions.

Based on the available evidence, which constitutes mostly on retrospective studies, the short-term use of HRT does not have any influence on the protective effect of the RRBSO on breast cancer incidence [1,27,28]. Women who are mutation carriers of BRCA 1 and BRCA2 genes may have different levels of cancer risk based on the differences of breast cancer subtypes they typically develop.

For BRCA1 mutation carriers, the use of HRT after RRBSO did not increase the incidence of breast cancer in a large prospective study [1]. On the contrary, an estrogen-only HRT may even have a protective effect against breast cancer. Although these figures did not reach statistical significance, Kostopoulos et al. recorded an 8% reduction in breast cancer risk (HR, 0.92; 95% CI, 0.83–1.01) for every year of estrogen replacement and an 8% increase in breast cancer risk (HR,1.08; 95% CI, 0.92–1.27) for every year of progestin replacement. In this study, the 10-year actuarial risk of breast cancer differed significantly between women using estrogen-only and combined estrogen and progestin replacement therapy (12% vs. 22%; absolute difference, 10%; *p*  =  0.04) and was particularly striking for women undergoing RRBSO prior to 45 years of age (9% vs. 24%; *p*  =  0.009). In this subgroup of women who underwent RRBSO prior to age 45, the protective effect on breast cancer risk conferred by an estrogen-only hormonal replacement therapy reached statistical significance with an 18% risk reduction (95% CI, 0.69–0.97) per year of treatment. In the same subgroup of women, a non-significant increase in breast cancer risk of 14% (95% CI, 0.90–1.46) was calculated for every year of combined estrogen-progestin replacement therapy. These findings are consistent with those reported in large prospective studies and randomized clinical trials performed in women who do not carry mutations of the BRCA genes [26,29,30,31,32].

The potential negative effect of the progestins on breast cancer risk may be explained by the role of the progesterone/receptor activator of nuclear factor-kB (RANK)-signaling pathway in BRCA 1 breast cancer development [33,34]. Although the molecular mediators and hormones associated with BRCA 1 and 2 mutations have not been clearly identified, progesterone clearly played a role in mammary tumorigenesis of BRCA 1/p53 in a murine model [35]. RANKL/RANK has also been found to be associated with progestin-driven mammary cancer in mouse models and may have a role in the etiology of BRCA1/2 mutation-driven breast cancer. Progesterone stimulates the secretion of RANKL/RANK by estrogen receptor (ER) and progesterone receptor (PR)-positive mammary epithelial cells, which in turn acts on ER/PR-negative epithelial progenitor cells, promoting proliferation and expansion of mammary epithelial cells. In BRCA1/2 carriers with triple-negative breast cancer, RANK has been implicated for the signaling on progenitor cells, which are believed to be “seed cells” and BRCA1 regulates the development of the human mammary stem and progenitor cell [35].

For BRCA 2 mutation carriers, available data on the use of HRT are less available. In these patients, the adoption of an HRT is probably less important given the later age at which they typically undergo an RRBSO and given their propensity to develop hormone receptor-positive breast cancers. Hence, HRT in this subset of patients should be adopted with caution.

In conclusion, the adoption of an estrogen-only hormonal replacement therapy after RRBSO does not increase the risk of breast cancer among women with a BRCA 1 mutation and should reassure BRCA1 mutation carriers considering risk-reducing surgery that HRT is safe. The potential adverse effect of progestin containing HRT warrants further studies. Similarly, studies should investigate whether route, regimen or dose of HRT differentially impacts breast cancer risk as well as the impact of intrauterine devices as HRT. The risk associated with progestins may be of little clinical impact in women undergoing both RRBSO and risk-reducing mastectomy. For women who select not to undergo a mastectomy, the use of progestins may be avoided in cases of previous hysterectomy or when a hysterectomy is performed in conjunction with the RRBSO.

Although the morbidity of a hysterectomy and bilateral salpingo-oophorectomy is somewhat higher than that of a bilateral salpingo-oophorectomy, women undergoing RRBSO, especially if aged under 45 years, should be counseled that the removal of the uterus will eradicate the need for progestins.
